# In this month’s Bulletin

**DOI:** 10.2471/BLT.23.000323

**Published:** 2023-03-01

In the editorial section, Hanna Teräs & Nellie Kartoglu (162) explain how civil society organizations contribute to emergency preparedness. Edgar Manuel Cambaza (163) proposes ways to expand the provision of nurses’ education.

Tatum Anderson (166–167) reports on efforts to improve diagnostics and treatments for invasive fungal pathogens. Rita Oladele talks to Gary Humphreys (168–169) about the need for more investment in invasive fungal pathogen surveillance, research and clinical capacity.

## Brunei, Cambodia, Indonesia, Lao People’s Democratic Republic, Malaysia, Myanmar, Philippines, Singapore 

### Vehicle safety interventions 

Jacobo Antona-Makoshi et al. (211–222) estimate potential death and disability averted.

## Cameroon

### Focusing cholera response activities

Jean Patrick Ouamba et al. (170–178) target prevention measures.

## China, Costa Rica, India, Indonesia, Mongolia, Peru, Philippines, United Republic of Tanzania, Viet Nam 

### Healthy weight in childhood 

Oliver Huse et al. (226–228) introduce a landscape analysis of factors contributing to obesity.

## India

### Towards tuberculosis elimination

Aakshi Kalra et al. (179–190) track case notifications.

### Accurate reporting of stillbirths and neonatal deaths 

Rakhi Dandona et al. (191–201) compare two sources of data.

## Uganda

### Cardiovascular abnormalities in children with pneumonia 

Eva Nabawanuka et al. (202–210) examine chest radiographs of hospitalised children.

## Viet Nam

### Reducing antimicrobial use in livestock

Juan J Carrique-Mas et al. (223–225) describe new legislation and challenges to full compliance.

